# Supplementary material for: Erythropoietin promotes hippocampal mitochondrial function and enhances cognition in mice
Source: Commun Biol. 2021 Aug 5;4:938. doi: 10.1038/s42003-021-02465-8 (PMC8342552; doi:10.1038/s42003-021-02465-8)
Supplement: Supplementary file 2 — Supplementary Information [file 42003_2021_2465_MOESM2_ESM.pdf]

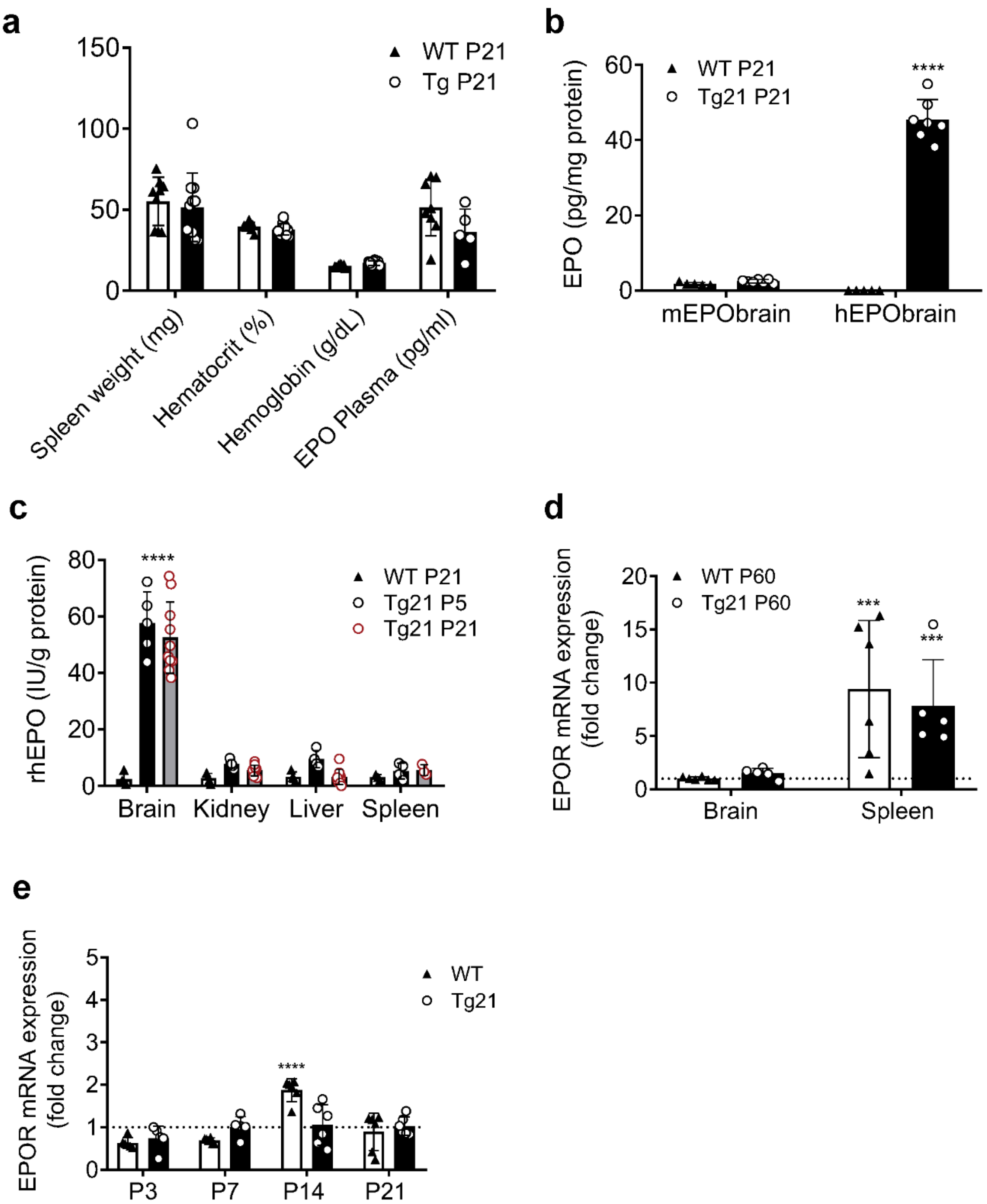

Supplementary Figure 1. Overexpression of recombinant human (rh)EPO in Tg21 mice is restricted to the brain. EPO receptors (EPOR) are 8-fold higher in spleen than in the brain and are not expressed in the cortex.

a) Measures of spleen weight (mg-ww), haematocrit (%), haemoglobin (g/dL), and plasma EPO concentration (pg/ml) at P21, showing no differences between genotypes; unpaired Student's t-test analyses; p = 0.99, spleen; p = 0.88, haematocrit; p = 0.07 haemoglobin; and p = 0.88, plasma EPO. **b)** Protein expression of murine (m)EPO and

rhEPO in the hippocampus at P21. Negligible expression of brain mEPO is detected in either genotype whereas rhEPO expression is significantly greater in Tg21 mice; 2-way ANOVA, $F(1,16) = 275$, ****$p < 0.0001$. **c)** Measures of rhEPO in brain, kidney, liver, and spleen in WT and Tg21 animals at P5 and in Tg21 at P21. rhEPO overexpression is primarily restricted to the brain, with a slight (~16% of respective measure in the brain) and transient expression in the liver of Tg21 mice at P5; 1-way ANOVA brain, $F(2,9) = 30.57$, ****$p < 0.0001$. **d)** EPOR mRNA expression in the brain and spleen of WT and Tg21 mice at P60, normalized to GAPDH. EPOR expression is 8-fold higher in the spleen (2-way ANOVA, $F(1,18) = 18.67$, ***$p = 0.0004$) with no difference between genotypes (2-way ANOVA, $F(1,18) = 0.1$, $p = 0.75$). **e)** EPOR mRNA expression in the cortex of WT and Tg21 mice across postnatal ages P: 3, 7, 14 and 21, normalized to GAPDH. Primarily, there is no change in EPOR expression with age or between genotypes. However, we did observe a transient increase in WT mice at P14; 2-way ANOVA, $F(1,38) = 2.77$, $p = 0.1$. Barplots with SD bars.

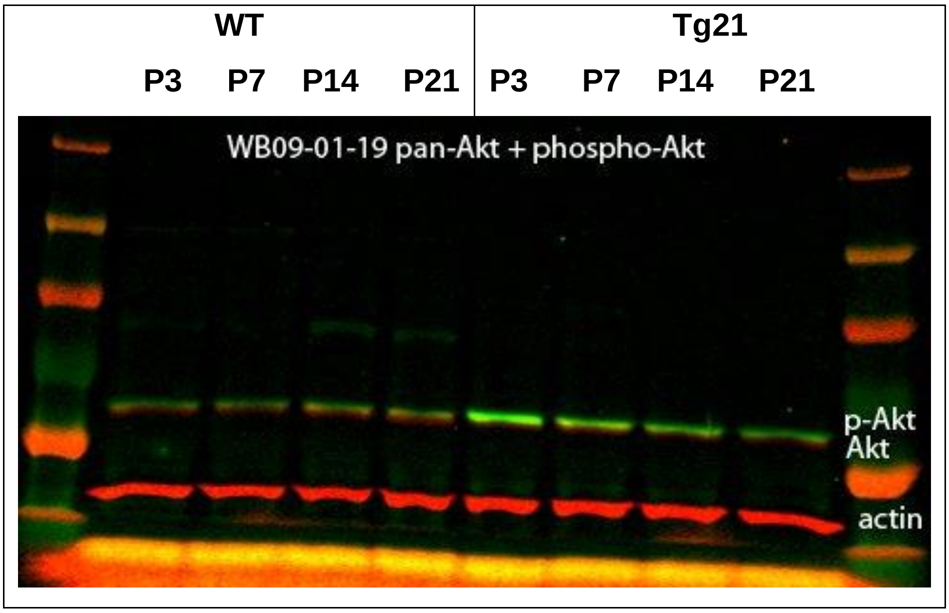

Supplementary Figure 2. Uncropped fluorescent Western blot gel of total AKT (red) and phosphorylated (green) protein expression in the hippocampus from WT and Tg21 mice across postnatal ages P: 3, 7, 14, 21. β-actin loading as control (red; lower bands). See figures 1k-i for statistics.

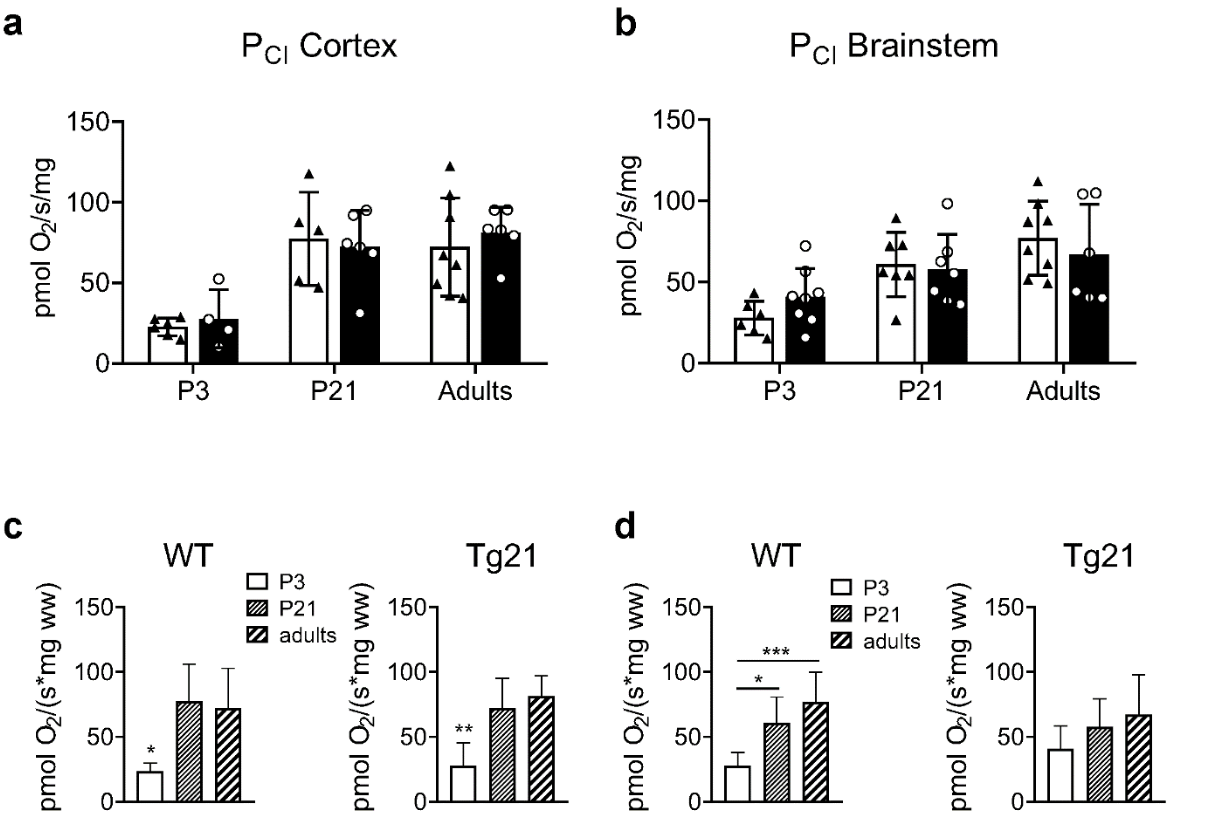

Supplementary Figure 3. EPO overexpression in the CNS does not influence mass-specific respiratory control in cortex and brainstem. Coupled respiration with maximal electron input specific to mitochondrial complex I (P_{CI}) throughout postnatal development in WT vs Tg21 mice in cortex (**a)** and brainstem (**b)**. No differences in P_{CI} oxygen consumption rates (OCR) were observed between genotypes. Cortex; 2-way ANOVA, $F(1,30) = 0.15$, $p = 0.7$. Brainstem; 2-way ANOVA, $F(1,36) = 0.0002$, $p = 0.99$. **c)** Cortex mass-specific respiration specific to complex I (P_{CI}) for WT and Tg21 mice at postnatal ages of P: 3, 21 and 60. An age dependent effect is observed in WT; 1-way ANOVA, $F(2,14) = 5.6$, *$p = 0.02$, and Tg21; 1-way ANOVA, $F(2,13) = 10.15$, **$p=0.002$. **d)** Brain stem mass-specific respiration specific to complex I (P_{CI}) for WT and Tg21 mice at postnatal ages of P: 3, 21, and 60. An age dependent effect is observed in WT; 1-way ANOVA, $F(2,18) = 11.55$, ***$p = 0.0006$; with multiple comparisons: *$p < 0.05$, **$p < 0.01$, ***$p < 0.001$. Barplots with SD bars.

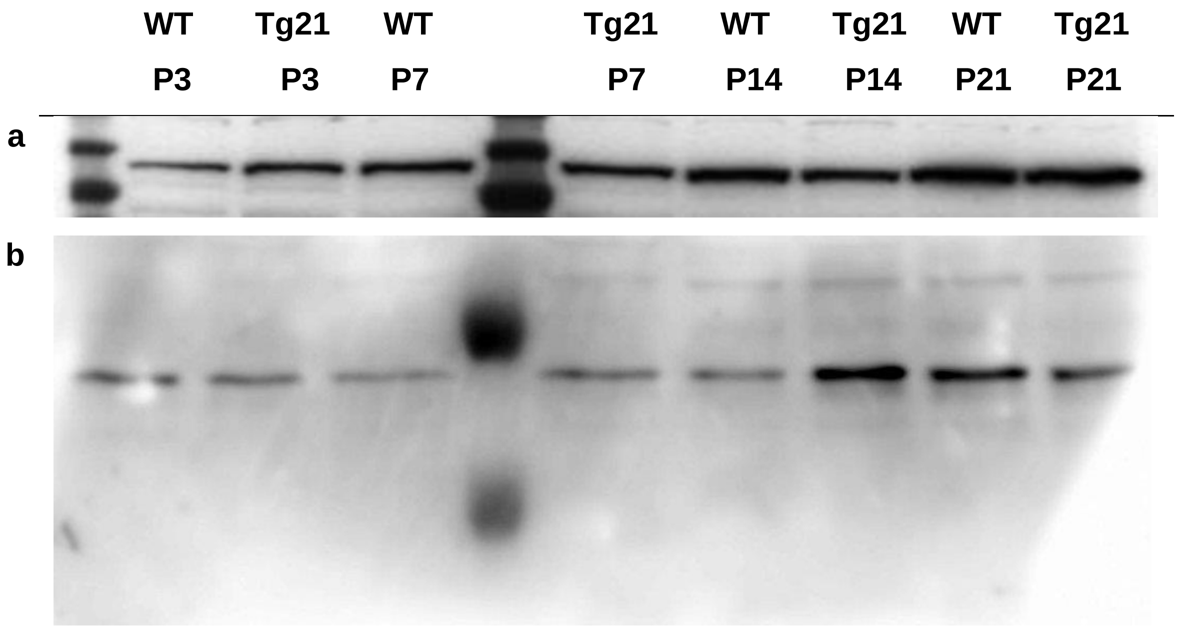

Supplementary Figure 4. Uncropped Western blot gel of voltage-dependent anion channel 1 (VDAC1) in WT and Tg21 mice across postnatal development. **a)** Vinculin as loading control. **b)** VDAC1 protein expression**.** See figure 4c for statistics.

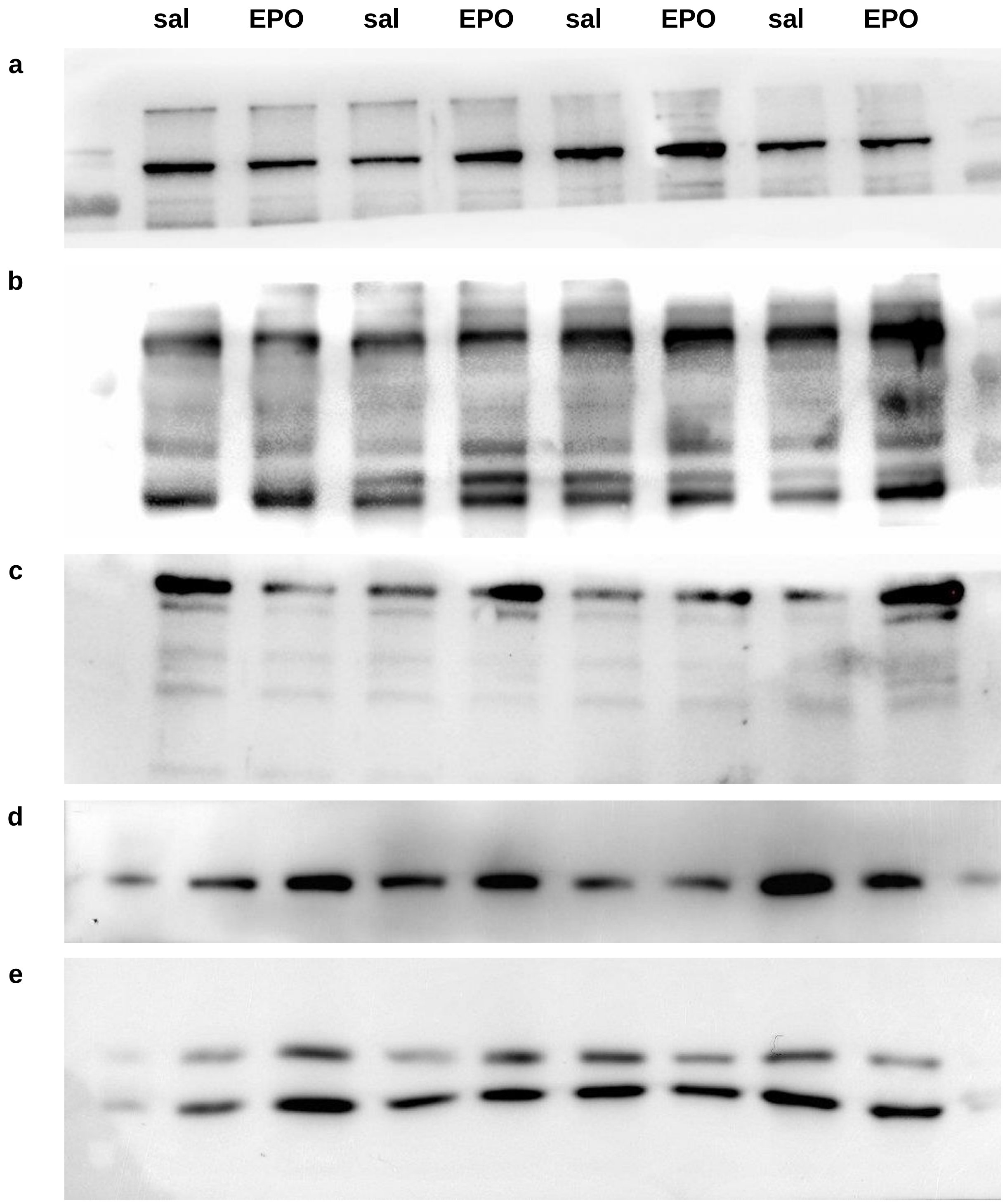

Supplementary Figure 5. Uncropped Western blot gels for AKT, Erk1/2 and VDAC1 after 3 days (P23-25) high dose intraperitoneal (i.p.) administration of EPO. a) Vinculin expression as loading control. **b)** Total AKT/Erk1/2; **c)** voltage-dependent anion channel 1 (VDAC1); **d)** pAKT and **e)** pErk1/2 expression at P26. See figure 5d,e,f,h and I for statistics.
