# Supplementary material for: Erythropoietin promotes hippocampal mitochondrial function and enhances cognition in mice
Source: Commun Biol. 2021 Aug 5;4:938. doi: 10.1038/s42003-021-02465-8 (PMC8342552; doi:10.1038/s42003-021-02465-8)
Supplement: Supplementary file 3 — Descriptions of additional Supplementary files [file 42003_2021_2465_MOESM3_ESM.pdf]

Description of Additional Supplementary Files

File name: Supplementary Data 1

Description: Data for Figure 1, Figure 3, Figure 5, Figure 6 and Figure 7. Captions are given at the top of each Excel file.
