# Supplementary material for: Erythropoietin promotes hippocampal mitochondrial function and enhances cognition in mice
Source: Commun Biol. 2021 Aug 5;4:938. doi: 10.1038/s42003-021-02465-8 (PMC8342552; doi:10.1038/s42003-021-02465-8)
Supplement: Supplementary file 5 — Reporting Summary [file 42003_2021_2465_MOESM5_ESM.pdf]

nature research

Corresponding author(s): Edith Marianne Schneider Gasser
edith.schneidergasser@uzh.ch

Last updated by author(s): 30.06.2021

Reporting Summary

Nature Research wishes to improve the reproducibility of the work that we publish. This form provides structure for consistency and transparency in reporting. For further information on Nature Research policies, see our Editorial Policies and the Editorial Policy Checklist.

Statistics

For all statistical analyses, confirm that the following items are present in the figure legend, table legend, main text, or Methods section.

n/a	Confirmed	
☐	☒	The exact sample size (*n*) for each experimental group/condition, given as a discrete number and unit of measurement
☐	☒	A statement on whether measurements were taken from distinct samples or whether the same sample was measured repeatedly
☐	☒	The statistical test(s) used AND whether they are one- or two-sided<br>*Only common tests should be described solely by name; describe more complex techniques in the Methods section.*
☐	☒	A description of all covariates tested
☐	☒	A description of any assumptions or corrections, such as tests of normality and adjustment for multiple comparisons
☐	☒	A full description of the statistical parameters including central tendency (e.g. means) or other basic estimates (e.g. regression coefficient) AND variation (e.g. standard deviation) or associated estimates of uncertainty (e.g. confidence intervals)
☐	☒	For null hypothesis testing, the test statistic (e.g. *F*, *t*, *r*) with confidence intervals, effect sizes, degrees of freedom and *P* value noted<br>*Give P values as exact values whenever suitable.*
☒	☐	For Bayesian analysis, information on the choice of priors and Markov chain Monte Carlo settings
☒	☐	For hierarchical and complex designs, identification of the appropriate level for tests and full reporting of outcomes
☐	☒	Estimates of effect sizes (e.g. Cohen's *d*, Pearson's *r*), indicating how they were calculated

Our web collection on statistics for biologists contains articles on many of the points above.

Software and code

Policy information about availability of computer code

Data collection	Hemoglobin analysis (Abbot Diagnostics Division, Santa Clara, CA, USA), RIA (Quanta Smart for Tri-Carb 4910TR, PerkingElmer); ELISA microplate reader (Photometer Asys DigiScan400, Software MIKROWIN 2000 4.11); RNA yield (Nanodrop 2000, ThermoFisher Scientific, USA), qRT-PCR (7500 Fast real-time PCR, ThermoFisher Scientific, Waltham, MA, USA); densitometry WB (Image J, NIH sofware and liCOR Odyssey Platform, Bioscences); highresolution respirometry (DatLab-Bioblast-O2k-Software, Innsbruck, Austria); WB fluorescence analysis (Li-COR Odyssey Plataform, Bioscences); EM imaging (100 kV transmission electron microscope,TEM - Philips CM100 and Telos); mitochondria and vesicles EM quantification (particle analysis plugin, Fiji, NIH); cognitive test tracking (Noldus EthoVision 1.96 system (Noldus Information Technology, Wageningen NL).
Data analysis	Prism 8.0.1., GraphPad Software, San Diego, CA, USA; Oligo Analyzer 3.1. IDT. Lubio Science.

For manuscripts utilizing custom algorithms or software that are central to the research but not yet described in published literature, software must be made available to editors and reviewers. We strongly encourage code deposition in a community repository (e.g. GitHub). See the Nature Research guidelines for submitting code & software for further information.

Data

Policy information about availability of data

All manuscripts must include a data availability statement. This statement should provide the following information, where applicable:

- Accession codes, unique identifiers, or web links for publicly available datasets
- A list of figures that have associated raw data
- A description of any restrictions on data availability

All data generated or analysed during this study are included in this published article (and its supplementary information files).

Field-specific reporting

Please select the one below that is the best fit for your research. If you are not sure, read the appropriate sections before making your selection.

[x] Life sciences [ ] Behavioural & social sciences [ ] Ecological, evolutionary & environmental sciences

For a reference copy of the document with all sections, see nature.com/documents/nr-reporting-summary-flat.pdf

Life sciences study design

All studies must disclose on these points even when the disclosure is negative.

Sample size	Sample size was determined with power anaylsis (sample size calculator) in which inter-individual variation (SD) of 30%, a p<=0.05 and a power (1-beta) of 0.9 was achieved. Initial samples were n=4, and according to the variance the final sample size was calculated assuming normal distribution. Our sample number was per age and genotype: n= 5-15 for blood, EPO and qEPOR measurements; n=4 for western blot analysis; n=6-8 for respirometry; n= 8, and n=10-18 for behaviour.
Data exclusions	Outliers would be removed after Grubb's tests calculator (Prism 9.0, GraphPad). No data falled into outlier criteria. Animals that explored less than 10 s each object in the NOR test were excluded.
Replication	We prove reproducibility of the data by introducing two different approaches: genetic overexpression of EPO in the brain and intraperitoneal injection of EPO. Both approaches showed similar impact of EPO in hippocampal metabolism.
Randomization	Pups from different breeding pairs were taken one after the other at each developmental age. At least six different breeding pairs per genotype were used for the study.
Blinding	Investigators were blinded during data collection

Reporting for specific materials, systems and methods

We require information from authors about some types of materials, experimental systems and methods used in many studies. Here, indicate whether each material, system or method listed is relevant to your study. If you are not sure if a list item applies to your research, read the appropriate section before selecting a response.

Materials & experimental systems

n/a	Involved in the study	
[ ]	[x]	Antibodies
[x]	[ ]	Eukaryotic cell lines
[x]	[ ]	Palaeontology and archaeology
[ ]	[x]	Animals and other organisms
[x]	[ ]	Human research participants
[x]	[ ]	Clinical data
[x]	[ ]	Dual use research of concern

Methods

n/a	Involved in the study	
[x]	[ ]	ChIP-seq
[x]	[ ]	Flow cytometry
[x]	[ ]	MRI-based neuroimaging

Antibodies

Antibodies used	Epo-Trac 1251 RIA kit, DiaSorin, Saluggia, Italy; rhEPO Quantikine IVD ELISA, R&D Systems; mEPO Quantikine ELISA, R&D systems; rabbit anti-p44/42 MAPK (Erk1/2) (Cell Signalling, #9102); rabbit anti-Phospho-p44/42 MAPK (p-Erk1/2) (Cell signalling, #9101); mouse anti-AKT(pan) (Cell Signalling, #9272); rabbit anti-Phospho-AKT(Ser473) (Cell Signalling, #9271); rabbit anti-VDAC1/Porin antibody (abcam, #ab15895); OxPhos Rodent WB Antibody Cocktail (ThermoFisher Scientific, #458099); rabbit anti-COX IV monoclonal antibody (3E11) (Cell Signaling, #4850).
Validation	All comercial antibodies are extensively used and sited. Here some sources: Epo-Trac 1251 RIA kit, DiaSorin, Saluggia, Italy (Glaus, T.M. et al (2004) Comp Biochem Physiol A Mol Integr Physiol, 138: 355-361); rhEPO Quantikine IVD ELISA, R&D Systems (Jelkmann, W. (1992) Physiol. Reviews 72:449); mEPO Quantikine ELISA, R&D systems (Bernaudin, M. et al. (2000) Glia 30:271); rabbit anti-p44/42 MAPK (Erk1/2) (Cell Signalling, #9102, Miaomiao Huo, et. al. (2020), J Cancer); rabbit anti-Phospho-p44/42 MAPK (p-Erk1/2) (Cell signalling, #9101, Singh H. et al (2020) J Neuroinflammation, 17:226); mouse anti-AKT(pan) (Cell Signalling, #9272, Jinchuan Chen, et. al. (2020), Mol Med Rep); rabbit anti-Phospho-AKT(Ser473) (Cell Signalling, #9271, Devi L. Ohno M. (2015), Tranl Psychiatry); rabbit anti-VDAC1/Porin antibody (abcam, #ab15895, Wang J. et al. (2020) Redox Biol 30:101415); OxPhos Rodent WB Antibody Cocktail (ThermoFisher Scientific, #458099, Kim H-J et al (2017) Sci Rep. 22,7(1):16041); rabbit anti-COX IV monoclonal antibody (3E11) (Cell Signaling, #4850, Anders P Mutvei, et. al (2020) Nat Commun).

Animals and other organisms

Policy information about studies involving animals; ARRIVE guidelines recommended for reporting animal research

Laboratory animals	C57Bl/6J (wild-type control (WT), and transgenic Tg(PDGFB-EPO)322Zbz, Tg21) mice from both gender at postnatal (P) ages: 3, 7; 14; 21, and 60 .
Wild animals	na
Field-collected samples	All mice were bred and housed under standard conditions at the University of Zurich. Animals were kept on a 12/12-h light/dark cycle with food and water provided ad libitum.
Ethics oversight	Animal experiments were performed following the ARRIVE guidelines and were approved by the Cantonal Veterinary Office of Zurich, Switzerland (ZH177_16).

Note that full information on the approval of the study protocol must also be provided in the manuscript.
